# Proliferative glomerulonephritis with monoclonal immunoglobulin deposition coexisting with ANCA-associated glomerulonephritis: a case report

**DOI:** 10.3389/fimmu.2025.1567277

**Published:** 2025-04-16

**Authors:** Yaqian Cheng, Yifan Zhang, Hui Chen, Yiyi Zheng, Zemin Wang

**Affiliations:** Department of Nephrology, Wenzhou Central Hospital, Wenzhou, China

**Keywords:** proliferative glomerulonephritis with monoclonal immunoglobulin deposition, monoclonal gammopathy of renal significance, ANCA-associated glomerulonephritis, corticosteroids, cyclophosphamide

## Abstract

Proliferative glomerulonephritis with monoclonal immunoglobulin deposition (PGNMID) is a rare variant of monoclonal gammopathy of renal significance (MGRS). Although the pathogenesis of PGNMID is not yet fully understood, it is currently hypothesized to originate from the intraglomerular deposition of pathogenic monoclonal immunoglobulins secreted by B cells or plasma cells. Typically, these deposits exhibit light chain restriction, with κ light chains being the most prevalent. Therapeutic strategies for PGNMID are based on targeting the abnormal or potential clone. However, the combination of corticosteroids and cyclophosphamide has also been reported. In this report, we describe an extremely rare clinical case of λ light chain type PGNMID occurring simultaneously with ANCA-associated glomerulonephritis. After treatment with the combination of corticosteroids and cyclophosphamide, the patient demonstrated reduced proteinuria and stable renal function.

## 1 Introduction

The concept of proliferative glomerulonephritis with monoclonal IgG deposition (PGNMID) was initially proposed by Nars (1) in 2004. Through the comprehensive analysis of numerous renal biopsy specimens, Nars identified 10 cases of immune-mediated glomerulonephritis that posed challenges in classification. Light microscopy revealed the characteristic features of proliferative glomerulonephritis within these cases. Immunofluorescence indicated the expression of a single light chain subtype (either κ or λ) and a single immunoglobulin heavy chain (IgG). Furthermore, electron microscopy uncovered granular electron-dense deposits in the mesangial, subendothelial, and subepithelial regions. Subsequently, Nars categorized these cases as PGNMID (1). PGNMID is recognized as one of the subtypes of monoclonal gammopathy of renal significance (MGRS), with κ light chain being the most prevalent subtype (2). The treatment of PGNMID is mainly based on interventions for abnormal clones or potential clones, usually with bortezomib-based regimens or rituximab-based regimens. However, the prognosis of patients with PGNMID is relatively poor. In Nasr’s research, it was found that the rate of progression to end-stage renal disease was 22% and the mortality rate was 16% (2). Given these concerning statistics, it is crucial that early diagnosis and prompt treatment are implemented to achieve a more favorable prognosis. However, with limited reports on PGNMID and clinicians’ insufficient understanding of this disease, the diagnosis and treatment of PGNMID are often delayed.

In this article, we describe a case of λ light chain PGNMID co-occurring with ANCA-associated glomerulonephritis. The purpose of this presentation is to expand the case database and improve clinicians’ knowledge of PGNMID.

## 2 Case report

A 33 - year - old unmarried, unemployed female patient was admitted to our hospital on October 5, 2023, presenting with “recurrent bilateral lower - limb edema for half a year.” Six months prior, she developed edema in both lower limbs without experiencing chest tightness, dyspnea, foamy urine, or fever. She visited the outpatient department of our hospital, where initial tests showed a serum creatinine level of 144 μmol/L, hemoglobin of 81 g/L, and a C - reactive protein (CRP) value of 50.3 mg/L. Urinalysis results indicated hematuria at 3 +, proteinuria at 3 +, a positive nitrite test, a leukocyte esterase result of +/-, 8 - 20 microscopic leukocytes per high - power field (HP), 31-40 microscopic erythrocytes per HP, 8 - 20 microscopic epithelial cells per HP, and bacteria (microscopic) at 1 +. At that time, based on these findings, the diagnosis was urinary tract infection, renal insufficiency, and proteinuria. She was treated with oral anti - infective medications and diuretics. However, the symptoms persisted with recurrent episodes. Recently, she revisited our hospital, and new tests revealed a serum creatinine level of 182 μmol/L, a serum albumin level of 25.7 g/L, and a hemoglobin level of 63 g/L. Urinalysis this time showed hematuria at 2 +, proteinuria at 2 +, with both nitrite and leukocyte esterase tests being negative. Subsequently, she was hospitalized for more in - depth diagnosis and treatment.

The patient had a history of two ophthalmic surgeries, but detailed information regarding these procedures was unavailable. She firmly denied a history of hypertension, diabetes, heart disease, cerebrovascular disease, and infectious diseases. Being unmarried and childless, her menstrual cycle was normal, and there was no family history of hereditary diseases. Moreover, she reported no history of smoking or alcohol use.

Upon admission, the physical examination showed a body temperature of 36.4°C, a pulse rate of 78 beats per minute, a respiratory rate of 17 breaths per minute, a blood pressure of 123/76 mmHg, and a body mass index (BMI) of 25 kg/m². The patient had an anemic appearance; her face was free of edema; the skin was dry, scaly, and had numerous scratch marks; and there was severe pitting edema in the lower limbs. Physical examinations of the cardiovascular, respiratory, and abdominal systems did not reveal any abnormal findings.

The laboratory investigations upon admission are presented in **Table 1**. Additionally, the patient underwent a comprehensive set of tests. These included antinuclear antibody spectrum analysis, serum protein electrophoresis, immunofixation electrophoresis, anticardiolipin antibody testing, Coombs test, anti - glomerular basement membrane antibody testing, tumor marker screening, and infectious disease screening. All these test results were negative. However, a significant discovery was the dual - positivity of p-ANCA (determined by indirect immunofluorescence assay) and MPO-ANCA (determined by immunoblotting). The cryoglobulin test was carried out after the patient resumed follow - up, and the result was negative. The electrocardiogram (ECG) showed sinus tachycardia, indicating an elevated heart rate without other rhythm disturbances. Echocardiography indicated left atrial enlargement, which might be associated with various factors such as increased cardiac filling pressure. A computed tomography (CT) scan of the chest revealed a nodule at the pleural margin of the horizontal fissure in the right middle and upper lobes. Abdominal CT showed cystic nodules in the bilateral adnexal regions, a calcification focus in segment 4 of the liver, and an enlarged spleen. However, the size and morphology of both kidneys were normal.

**Table 1:** Laboratory investigation upon admission and during the latest follow - up.

Parameters	Value (upon admission)	Value (the latest follow- up)	References	Unit
White blood cell count	7.5	7.3	3.5-9.5	×10^9^/L
Red blood cell count	2.53	2.87	3.8-5.1	×10^12^/L
Hemoglobin	63	80	115-150	g/L
Platelet	250	160	125-350	×10^9^/L
Mean corpuscular hemoglobin	24.9	27.9	27.0-34.0	pg
Mean corpuscular volume	84.6	89.5	82.0-100	fL
Mean corpuscular hemoglobin concentration	294	311	316-354	g/L
Serum albumin	25.7	37.4	40.0-55.0	g/L
Serum globulin	44.6	34.3	20.0-40.0	g/L
Serum creatinine	182	262	41-73	μmol/L
eGFR	31	19.7	>90	mL/min
Serum uric acid	496	508	155-357	μmol/L
Glucose	5.1	4.9	3.9-6.1	mmol/L
Potassium	4.13	4.81	3.5-5.3	mmol/L
Calcium	2.05	2.13	2.11-2.52	mmol/L
Phosphorus	1.21	0.93	0.85-1.51	mmol/L
Serum iron	3.2	5.2	7.8-32.2	μmol/L
Total cholesterol	3.15	–	2.84-5.69	mmol/L
Triglycerides	1.17	–	0.56-1.70	mmol/L
Erythrocyte sedimentation rate	20	–	0-20	mm/h
IgG	20.19	–	8.6-17.4	g/L
IgA	3.51	–	1.00-4.20	g/L
IgM	3.52	–	0.50-2.80	g/L
Complement C3	0.74	–	0.70-1.40	g/L
Complement C4	0.19	–	0.10-0.40	g/L
IgG4	4.55	–	0.03-2.01	g/L
24h urinary protein	2.98	–	0-0.15	g
Urine albumin/creatinine ratio	1877.6	783.3	0-30.0	mg/g

**Figure 1:**
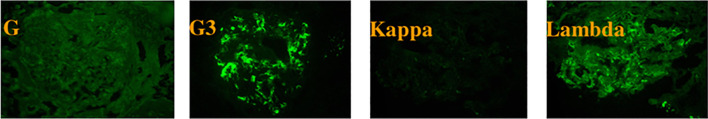
Immunofluorescence microscopy reveals granular deposits of IgG3 and λ light chains in the mesangial region of the glomerulus.

**Figure 2:**
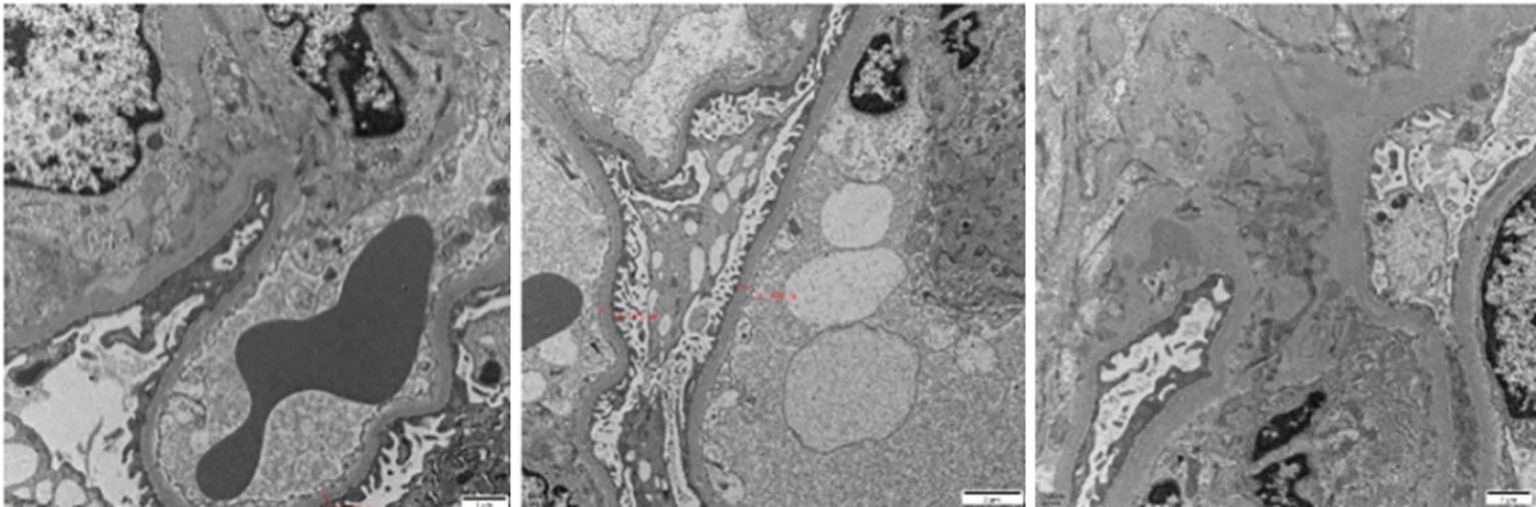
Electron microscopy reveals electron-dense deposits in the mesangial region of the glomerulus.

**Figure 3:**
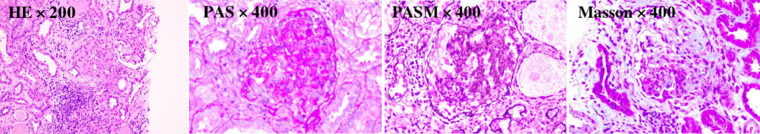
The mesangial cells and matrix in the glomerular mesangial area exhibit mild to moderate proliferation. The wall of the renal capsule is thickened, and crescent formation can be observed. The renal tubulointerstitium shows acute lesions on the basis of chronic changes.

Renal biopsy, a pivotal diagnostic procedure, was carried out on October 9, 2023. Light microscopy of the renal tissue specimen showed that out of 16 glomeruli, 8 were globally sclerotic, indicating advanced glomerular damage, and 3 had segmental sclerosis. In the remaining glomeruli, there was mild - to - moderate mesangial cell and matrix proliferation, along with crescent formation. Tubular changes included vacuolar and granular degeneration of tubular epithelial cells, accompanied by multifocal atrophy, reflecting tubular injury. The renal interstitium exhibited multifocal lymphocytic and mononuclear cell infiltration, as well as focal plasma cell infiltration, indicating an inflammatory process. Moderate fibrosis was observed in the cortical interstitium, and moderate - to - severe fibrosis was present in the medullary interstitium. Congo red staining was negative, ruling out amyloidosis. Immunofluorescence analysis demonstrated diffuse and granular deposits of IgG (+/to +), IgA (+/-), IgM (+/-), C3 (+++), C1q (+++), λ (++ to +++), κ (+/-), IgG3 (+++), and IgG4 (-) in the mesangial area. These specific immunoglobulin and complement deposits are highly indicative of proliferative glomerulonephritis with monoclonal immunoglobulin deposition (PGNMID). Electron microscopy showed that most capillary loops were twisted and wrinkled, with mesangial cell and matrix proliferation in the glomerular mesangial area. The majority of the remaining podocyte foot processes were fused. Electron - dense deposits were detected in the segmental mesangial areas of the glomeruli. Renal tubular epithelial cells showed vacuolar degeneration, and there was inflammatory cell infiltration and collagen fiber proliferation in the renal interstitium (**Figures 1**–**3**).

Based on the above - mentioned comprehensive examinations and in - depth renal pathology findings, the patient was diagnosed with the following conditions: 1) Proliferative glomerulonephritis with monoclonal immunoglobulin deposition (λ light - chain type). The diagnosis was established based on the characteristic renal biopsy findings, including the specific pattern of immunoglobulin deposition and glomerular changes. 2) ANCA - associated glomerulonephritis. The dual positivity of p - ANCA and anti - myeloperoxidase antibodies, along with the renal biopsy - proven crescent formation, supported this diagnosis. 3) Chronic kidney disease stage 3, determined by the estimated glomerular filtration rate calculated from the serum creatinine level and other relevant factors. 4) Moderate anemia, as indicated by the low hemoglobin level. 5) Hypoalbuminemia, diagnosed based on the decreased serum albumin level. 6) Sinus tachycardia, evident from the ECG results. 7) Pulmonary nodule, detected by the chest CT scan.

The patient was initially treated with intravenous methylprednisolone at a dose of 500 mg per day for 3 consecutive days. This high-dose intravenous corticosteroid was administered to swiftly suppress the immune response. Subsequently, the patient was prescribed oral prednisone at a daily dose of 60 mg to maintain the anti-inflammatory effect. Additionally, the treatment was combined with intravenous cyclophosphamide at 0.8 g, which was given every two weeks for a total of two doses. The choice of cyclophosphamide and its dosing regimen was based on the patient’s overall condition, including the severity of the disease and renal function. Supportive therapies included calcium supplementation to prevent corticosteroid induced osteoporosis, gastric protection to reduce the risk of gastrointestinal ulcers due to corticosteroid use, albumin supplementation to correct hypoalbuminemia, and correction of anemia using roxadustat capsules and polysaccharide iron complex capsules. At the one - month follow - up visit, the patient’s bilateral lower - limb edema had subsided, and the 24 - hour urine albumin level had decreased to 1.89 g. Renal function remained stable. Considering these positive responses, the prednisone dose was reduced to 50 mg/day, and a single dose of intravenous cyclophosphamide at 0.6 g was administered. Since then, the patient was lost to follow - up. In March 2025, were - established contact with the patient. The patient claimed that during the period of being lost to follow - up, she had no discomfort and had not undergone any examinations or treatments. Subsequently, we conducted a comprehensive laboratory investigation of her. The new test indicates that MPO has turned negative, and the urine albumin - creatinine ratio has decreased to 783.3mg/g. Other test results are presented in **Table 1**.

## 3 Discussion

PGNMID is a rare disease, posing a challenge to clinicians and researchers in the field of nephrology. The average age of onset is 55 years old, and among patients over 20 years old, PGNMID represents only 0.8% of renal biopsies (3). The clinical manifestations of PGNMID lack specificity. As has been reviewed in relevant articles, patients typically present with nephrotic syndrome, which commonly manifests as massive proteinuria and edema. Additionally, some patients also experience hematuria, with only a small proportion showing gross hematuria (4). Renal insufficiency is observed in approximately two-thirds of the patients; however, it rarely presents as rapidly progressive glomerulonephritis. Furthermore, less than 10% of the patients require dialysis at the time of diagnosis (5). Hypocomplementemia is infrequent, with only 20% of patients showing reduced serum complement C3 and C4 levels. Additionally, cryoglobulin tests yield negative results, and the detection rates of monoclonal immunoglobulins (mIg) in both serum and bone marrow are low (6, 7). Renal biopsy is of crucial importance for the diagnosis of PGNMID. Under light microscopy, the main findings are proliferative changes, including membranoproliferative glomerulonephritis (MPGN) and mesangial proliferative glomerulonephritis (MsPGN). In a minority of cases, membranous nephropathy changes are observed, and 10-50% of patients display crescent formation (4). Immunofluorescence reveals monoclonal deposits restricted to the glomeruli, with a granular distribution in the mesangium and glomerular capillary walls. The most commonly detected immunoglobulin is IgG, whereas IgM and IgA are observed less frequently, accounting for only 10% of PGNMID cases (6). Among IgG subtypes, IgG3 is the most prevalent, comprising 60-68% of cases (2, 7). The deposits are typically light-chain-restricted, with 73% of patients showing κ monotypic expression (2). Additionally, C3 deposition is commonly found in the glomeruli, and C1q deposition is detected in 55-64% of cases (2, 7). On electron microscopy, the deposits appear granular and are distributed within the mesangium and subendothelium.

In this clinical case, the patient presented with recurrent bilateral lower - limb edema. Immediately, a comprehensive battery of ancillary tests was commenced to facilitate the diagnostic process. Initially, the serum immunofixation electrophoresis test resulted in a negative finding, while the serum protein electrophoresis demonstrated no aberrant electrophoretic patterns. These outcomes play a pivotal role in ruling out hematological malignancies, including multiple myeloma and Waldenström’s macroglobulinaemia. The patient’s tumor markers, such as carcinoembryonic antigen (CEA), alpha - fetoprotein (AFP), and cancer antigen 125 (CA125), were all determined to be within the normal reference range. Subsequently, a comprehensive whole - body CT scan was meticulously performed, which did not disclose any indication of tumors. These results significantly constricted the scope of potential differential diagnoses, effectively guiding the diagnostic workup toward non - neoplastic etiologies. The antinuclear antibody (ANA) test, in conjunction with the assays for anti - double - stranded DNA (anti - ds - DNA) antibody and anti - Smith (anti - Sm) antibody, all yielded negative outcomes. Moreover, the complement levels were ascertained to be within the normal reference intervals. Collectively, these results effectively excluded the possibility of lupus nephritis. Following the comprehensive battery of preceding tests, a renal biopsy was carried out. The biopsy findings demonstrated proliferative glomerulonephritis. Significantly, granular deposits of IgG3 and λ light - chain were detected within the glomerular mesangial area, with concomitant deposits of C3 and C1q in the glomeruli. These specific immunohistochemical and ultrastructural manifestations not only buttress the diagnosis of proliferative glomerulonephritis but also offer vital clues for discerning it from other glomerular pathologies.

In the context of differential diagnosis, several conditions required meticulous consideration and exclusion. Lymphocytic leukemia, associated with abnormal proliferation of lymphocytes in the blood and bone marrow along with characteristic cytogenetic and molecular abnormalities, was absent in this case. In terms of cryoglobulinemia, considering the lack of typical manifestations such as purpura and Raynaud’s phenomenon, as well as the negative result of the cryoglobulin test, this diagnosis is excluded. Light chain deposition disease, which typically involves the deposition of monoclonal light chains predominantly within the tubular basement membranes and blood vessel walls, was excluded owing to the distinct deposition pattern in contrast to the glomerular deposits observed in this biopsy. Fibronectin glomerulopathy, characterized by distinct histological features, such as the presence of abundant fibronectin in the glomeruli, was not detected in the current renal biopsy. Considering the totality of the patient’s clinical manifestations, laboratory test results, and the detailed pathological findings from the renal biopsy, a diagnosis of PGNMID was suggested. These comprehensive data, collectively, served as a strong diagnostic foundation, aligning relatively well with the established diagnostic criteria for PGNMID.

In certain cases, patients diagnosed with PGNMID have been observed to concomitantly exhibit a variety of other conditions, such as solid tumors, autoimmune diseases, myelodysplastic syndromes (MDS), and primary renal amyloidosis (4). Nevertheless, the precise nature of the direct association between PGNMID and these comorbidities remains elusive. A comprehensive search of multiple international databases revealed no previously reported cases of PGNMID co - occurring with ANCA - associated glomerulonephritis. Consequently, this case represents the first reported instance of such a combination. PGNMID and ANCA - associated glomerulonephritis exhibit distinct pathogenic mechanisms and pathological manifestations. Specifically, the hallmark characteristic of PGNMID is the deposition of pathogenic monoclonal immunoglobulin. In contrast, ANCA - associated glomerulonephritis is distinguished by pauci-complex deposition. Given these differences, the specific reasons underlying the co - occurrence of these two diseases demand further in - depth investigation. Moreover, studies have demonstrated that patients diagnosed with ANCA - associated glomerulonephritis who also present with immune - complex deposition are prone to experience more severe proteinuria, elevated levels of serum creatinine, and a higher rate of crescent formation. These factors are associated with a less favorable prognosis (8). This shows the critical importance of implementing optimal management strategies for such patients to ensure better clinical outcomes.

Although the precise pathogenesis of PGNMID remains unclear, it is commonly hypothesized that it results from the deposition of nephrotoxic monoclonal immunoglobulins (mIg) produced by B cells or plasma cells within the glomeruli. Consequently, current therapeutic strategies for PGNMID are predominantly guided by the identification of a clone or the indication of a potential clone. Specifically, in cases where a plasma cell clone is identified, bortezomib-based chemotherapy is recommended. Conversely, for patients presenting a lymphocytic clone, anti-CD20 monoclonal-antibody-based treatment is suggested (4). Additionally, scholars have treated patients with PGNMID without a detectable clone using rituximab or bortezomib, under the assumption of a potential clone, and favorable clinical responses have been demonstrated in these patients (9). It is worth emphasizing that other medications, including renin-angiotensin-system (RAS) inhibitors, corticosteroids, and cyclophosphamide, may also possess therapeutic effects. For patients without nephrotic syndrome, those in chronic kidney disease (CKD) stages 1-2, or those with PGNMID accompanied by viral infection, RAS inhibitors can be considered as a monotherapy option (10). In cases where PGNMID patients display pathological manifestations of MsPGN and advanced glomerulosclerosis, corticosteroid treatment has yielded promising results (11). Additionally, the combination of corticosteroids and cyclophosphamide has also attained therapeutic efficacy (12). Furthermore, reports indicate that both daratumumab and the combination of lenalidomide with dexamethasone can effectively induce renal remission in patients with PGNMID (13, 14).

Regarding the treatment of ANCA associated glomerulonephritis, induction-remission protocols commonly advocate the combination of corticosteroids with either rituximab or cyclophosphamide. For patients with MPO-ANCA-associated vasculitis, the induction-remission rates of rituximab-based regimens and those based on cyclophosphamide are comparable (15). However, cyclophosphamide has been demonstrated to be more effective in enhancing short-term renal outcomes (16). Given the insufficient evidence regarding the use of rituximab in patients whose serum creatinine levels exceed 350 μmol/L, the Kidney Disease: Improving Global Outcomes (KDIGO) guidelines recommend that patients with severe ANCA associated glomerulonephritis should be treated with the classical regimen consisting of corticosteroids combined with cyclophosphamide (17). Therapeutic plasma exchange serves as an adjunctive therapy for patients with ANCA-associated vasculitis, capable of rapidly eliminating autoantibodies from the bloodstream. Nevertheless, when implementing this therapeutic measure, it is imperative to meticulously weigh the benefits against the risks. A randomized controlled study spanning seven years has demonstrated that in patients with ANCA-associated vasculitis whose eGFR<50 ml/minute/1.73 m², plasma exchange fails to reduce the incidence of either patient mortality or end-stage kidney disease (ESKD) (18). Moreover, plasma exchange is associated with an elevated risk of severe infections (19). Given that the patient’s glomerular filtration rate was at a relatively low level and she had already been on a treatment regimen of glucocorticoids combined with cyclophosphamide, we did not recommend plasma exchange for the patient. The pathogenesis of ANCA-associated vasculitis involves the activation of the alternative complement pathway, leading to the production of C5a (20). Avacopan inhibits the activation of neutrophils by targeting the C5a receptor (20, 21). Multiple studies indicate that Avacopan can be safely applied to patients with ANCA - associated vasculitis to enhance the long-term remission rate and improve renal function (22–24). However, during the patient’s treatment period, Avacopan had not been approved for marketing in China.

In this particular case, the patient was diagnosed with PGNMID complicated by ANCA - associated glomerulonephritis. The glucocorticoid - cyclophosphamide combination regimen was selected as it is suitable for both scenarios. After one month of treatment, the patient’s edema resolved, proteinuria decreased, and renal function remained stable. Despite the absence of data during the period of loss to follow - up, the latest laboratory findings reveal an elevation in the levels of albumin and hemoglobin, along with a remarkable reduction in urinary protein. These fully demonstrate the efficacy of the established treatment regimen. However, the elevation of serum creatinine is considered to be associated with the poor renal pathological type and the interruption of treatment. In contrast, another young patient with PGNMID showed a poor response to the treatment with glucocorticoids and cyclophosphamide. Despite undergoing treatment for as long as four years, she still developed nephrotic syndrome. Eventually, remission was achieved after she received a combination therapy regimen of bortezomib, cyclophosphamide, and dexamethasone (25).

Nevertheless, this case has some limitations. Firstly, more comprehensive diagnostic evaluations, such as bone marrow aspiration, would have been beneficial for our case study. However, the patient refused this measure as it is an invasive procedure with a high cost. Secondly, it is regrettable that the patient was lost to follow-up one month after the initiation of treatment. During this period, any information about the patient was missing. Complete follow - up data will make this case more comprehensive.

In summary, this case report presents a rare instance of λ light-chain PGNMID complicated by ANCA - associated glomerulonephritis, representing the first such case reported worldwide. PGNMID is one of the least understood entities within the spectrum of MGRS. As more cases are reported and studied, the enigma surrounding PGNMID will gradually be unraveled. Currently, our understanding of PGNMID is steadily progressing. In the future, the emergence of new technologies and methodologies is anticipated to further enhance its detection and treatment outcomes.

## Data Availability

The original contributions presented in the study are included in the article/supplementary material. Further inquiries can be directed to the corresponding author.
